# Five Tips for Teaching and Learning During the COVID-19 Movement Control Order Era: A Family Medicine Perspective

**DOI:** 10.21315/mjms2020.27.6.15

**Published:** 2020-12-29

**Authors:** Malanashita Ganeson, Sasikala Devi Amirthalingam, Kwa Siew Kim

**Affiliations:** Department of Family Medicine, International Medical University Clinical Campus Seremban, Negeri Sembilan, Malaysia

**Keywords:** teaching, learning, COVID-19, primary healthcare, education

## Abstract

The Malaysian government’s ongoing movement control order (MCO) to prevent coronavirus disease 2019 (COVID-19) spread, has disrupted the teaching and learning (T&L) activities of higher education institutions in the country. This paper seeks to outline the steps taken by the Department of Family Medicine of the International Medical University (IMU), Malaysia, to adapt its online teaching and learning activities. The five tips are: i) understand how to use online T&L platforms; ii) teachers should create multiple communication channels; iii) ensure attendance is captured; iv) enhance the online T&L experience and v) conduct online formative assessments.

## Introduction

Given the rising number of coronavirus Disease 2019 (COVID-19) cases within Malaysia, the government announced an unprecedented movement control order (MCO) on 16 March 2020, which was to last from 18–31 March 2020 (1). The MCO was subsequently extended (2–4) and it remains in effect at the time of writing. The order resulted in the closure of all higher education institutions, forcing the International Medical University (IMU), Malaysia, to fully transition to online teaching and learning (T&L) activities. This paper shares the experiences of the Department of Family Medicine in adapting to those changes.

## Teaching and Learning Before the Movement Control Order Period

Prior to the MCO, the Department of Family Medicine employed a blended learning approach. The existence of an e-learning portal (IMU E-Learn portal) enabled the online provision of student handbooks, task-based learning (TBL) guides, reading materials, interactive lectures, quizzes and discussion boards. These resources were complemented by face-to-face teaching sessions comprised of clinical teaching at government primary healthcare clinics otherwise known as *Klinik Kesihatan* (KK), case-based discussion (CBD), TBL, mentor-mentee session (MMS) and procedural skills session. Students also engaged in a one-week general practice (GP) posting to expose them to the Malaysian private healthcare sector. Attendance was captured either manually or using quick response (QR) codes. Assessment within the posting rotation was formative in nature, consisting of logbooks, case summaries, GP reports, evidence-based medicine (EBM) commentaries, a mid-posting clinical exam and an end-of-posting clinical exam.

## Challenges During the Movement Control Order

To comply with the MCO’s regulations, all face-to-face T&L activities were suspended or converted to online sessions. The first challenge brought on by the MCO was deciding which sessions to move online and which to postpone until after the MCO had been lifted. KKs are the first line of contact for many patients and conducting T&L activities at these centres would have put teachers, students and patients at risk of COVID-19 infection. Thus, all clinical teaching was put on hold. This decision led to the second challenge, namely, maintaining student engagement and motivation while ensuring that students retained their practical knowledge and clinical skills. This challenge was complicated by the need to rapidly and smoothly transition to online T&L (5) to ensure minimal disruption to the students’ learning experience. Other challenges included capturing attendance and converting assessment modalities to online formats.

## Adapting Teaching and Learning and Overcoming Challenges: Tips and Experiences

### Tip 1: Understand How to Use Online T&L Platforms

Universities should ensure that students and teachers are familiar with online T&L platforms to help ease the transition process (6). Within our institution, staff and students are familiar with E-Learn portal and the portal already contained resources for various T&L sessions. Hence, we continue to use this modality for asynchronous sessions. However, the IMU E-Learn portal lacks features for synchronous sessions, necessitating the use of an alternative platform. Therefore, we chose the Microsoft Teams platform for our synchronous sessions because it is readily available.

Various free online platforms and social media applications can facilitate the delivery of asynchronous and synchronous T&L activities (7). Platforms, such as Microsoft Teams and Google Classroom, act as virtual classrooms; synchronous or asynchronous T&L sessions can be conducted and materials can be shared. Sound knowledge about how to use these platforms is essential to enhance the user experience, reduce frustration and ensure the smooth execution of T&L activities. Providing staff and students with basic user guides for these platforms is essential. Useful information for guides includes how to create classrooms/teams, schedule/record/execute classes and share materials. Guides should ideally be sent to staff and students before the commencement of the family medicine posting. Additionally, staff and students who are savvy in information technology, can act as reference points for those having difficulty using the platforms.

### Tip 2: Teachers Should Create Multiple Communication Channels

Ideally, student engagement should be done on a minimum of two platforms: one for T&L and another for direct communication. In addition to Microsoft Teams, we are utilising WhatsApp for direct communication.

Some T&L platforms incorporate direct communication features; however, it is best if the direct communication channel is independent from the T&L platform to ensure that communications are maintained if either platform experiences a disruption. Direct communication platforms include WhatsApp, Telegram and Facebook Messenger; they are easily accessible and widely utilised through smartphones. Users of these platforms are able to respond quickly, enabling the easy scheduling of classes and the dissemination of important information. Should synchronous sessions be disrupted, users can shift to asynchronous sessions through these platforms (7).

### Tip 3: Ensure Attendance is Captured

We captured attendance using the Microsoft Teams platform as it has a built-in attendance feature. We then manually upload the attendance records to an online attendance sheet, which is maintained by administrative personnel.

Tracking students’ actual activities during sessions can be a challenge because some students might log in and then leave while others might engage in other activities while logged into a session. Hence, online T&L places extra responsibility on students compared to face-to-face T&L, emphasising self-motivation and integrity. Specific steps that can be incorporated into sessions to help curb undesirable behaviours are explained in Tip 4.

### Tip 4: Enhance the Online T&L Experience

We moved TBLs, CBDs and MMSs online but postponed clinical teaching and procedural sessions, including GP postings. All online sessions are timetabled and reminders are sent 15 min before the commencement of each session.

Synchronous online sessions are conducted using Microsoft Teams. TBL sessions consisting of groups of more than 30 students are conducted almost daily and lasts 2 h–3 h. Weekly MMSs and CBDs are conducted in groups of four to five students, and each session lasts 1 h–2 h.

The teachers decide how best to conduct the sessions. One strategy is the flipped classroom method (6). Topic-specific assignments, reading materials, online videos and pre-recorded lectures are provided to students using the IMU E-Learn portal or Microsoft Teams, enabling the students to access the materials at their convenience and prepare for the lessons. The synchronous online teaching sessions focuses on discussions related to the assigned topics. In addition, gamification strategies like Kahoot are used to create quizzes for discussion. Students are encouraged to ask questions and make presentations during these sessions. Another strategy is for teachers to question students at random thus allowing for informal attendance checks. These strategies allow teachers to maintain student engagement.

One strategy adopted to preserve students’ clinical skills involves teachers playing the role of simulated patients and students taking their history. Physical examination and other practical skills are discussed. This strategy aims to maintain history taking and clinical reasoning skills as well as theoretical knowledge of physical examination and practical skills.

In the face of the COVID-19 pandemic, many countries are adapting and advancing the use of telehealth services due to the numerous clinical and socioeconomic advantages in addition to the ability of telehealth services to reduce the spread of communicable diseases (8). Teaching students consultation skills online exposes them to telehealth. The use of trained simulated patients can enhance the value of such sessions.

We acknowledge the challenges of teaching physical examination and procedural skills online and find it quite impossible. However, we may use online resources to activate student’s prior knowledge and facilitate demonstration and explanations of tasks (6). Hands on training may be left for a suitable time later.

We ensure that time is allocated for self-directed learning to allow students to reflect and access asynchronous T&L activities. Additionally, we developed some rules to enhance student engagement and accessibility during synchronous sessions. For example, recording of all online synchronous sessions is made mandatory to aid students who are unable to attend or actively participate due to internet connectivity issues. Intermittent usage of video and chat functions are encouraged to promote engagement. Having users mute their microphones unless they are speaking, helps reduce environmental noise.

### Tip 5: Conduct Online Formative Assessments

We encourage the use of online formative assessments to keep students motivated. However, our existing assessment formats (logbooks, case summaries and EBM commentaries) are tied to real patient encounters and as such are best left for a time when clinical teaching resumes. Considering this a setback, we advocate the use of alternative modes of formative assessments along with modification of pre-existing methods. For example, as noted above, we continue to conduct clinical exams but shifted to an online format in which students take history from teachers who played simulated patient roles to enable the assessment of their history taking skills.

The topic of online assessments needs to be further explored and enhanced. Medical schools are already adapting to online assessments, as seen by the bold step taken by Imperial College London to conduct final year medical exams online (9).

## Summary

Currently, questionnaires are being administered to IMU staff and students to assess the effectiveness and acceptability of our online T&L activities. T&L during the MCO has been a challenging yet eye-opening experience that has taught us the value of crisis preparedness. As this pandemic has yet to end, it is likely that online T&L will become the norm. Given the possibility of future pandemics, adapting to online T&L is a matter of necessity and urgency.
